# A case report of Achilles tendon distractive rupture after shock wave therapy

**DOI:** 10.1093/jscr/rjaf206

**Published:** 2025-04-05

**Authors:** Evangelia Argyropoulou, Evangelos Sakellariou, Panagiotis Karampinas, Meletis Rozis, Athanasios Galanis, Ioannis Kolovos, Panagiotis Antzoulas, Angelos Kaspiris, Elias Vasiliadis, John Vlamis, Spiros Pneumaticos

**Affiliations:** Department of Orthopaedics and Traumatology, University General Hospital of Patras, 26504 Rion 1, Patras, Greece; Department of Orthopaedic Surgery, KAT Attica General Hospital, National & Kapodistrian University of Athens, 14561 Nikis 2, Athens, Greece; Department of Orthopaedic Surgery, KAT Attica General Hospital, National & Kapodistrian University of Athens, 14561 Nikis 2, Athens, Greece; Department of Orthopaedic Surgery, KAT Attica General Hospital, National & Kapodistrian University of Athens, 14561 Nikis 2, Athens, Greece; Department of Orthopaedic Surgery, KAT Attica General Hospital, National & Kapodistrian University of Athens, 14561 Nikis 2, Athens, Greece; Department of Orthopaedic Surgery, KAT Attica General Hospital, National & Kapodistrian University of Athens, 14561 Nikis 2, Athens, Greece; Department of Orthopaedics and Traumatology, University General Hospital of Patras, 26504 Rion 1, Patras, Greece; Department of Orthopaedic Surgery, KAT Attica General Hospital, National & Kapodistrian University of Athens, 14561 Nikis 2, Athens, Greece; Department of Orthopaedic Surgery, KAT Attica General Hospital, National & Kapodistrian University of Athens, 14561 Nikis 2, Athens, Greece; Department of Orthopaedic Surgery, KAT Attica General Hospital, National & Kapodistrian University of Athens, 14561 Nikis 2, Athens, Greece; Department of Orthopaedic Surgery, KAT Attica General Hospital, National & Kapodistrian University of Athens, 14561 Nikis 2, Athens, Greece

**Keywords:** Achilles tendon, Achilles tendinopathy, Achilles rupture, shock wave therapy

## Abstract

The primary objective of this research essay is to critically examine the relationship between shock wave therapy usage and subsequent. Achilles tendon ruptures, with a specific focus on a case involving a 68-year-old patient with a history of Achilles tendinopathy, who experienced a perceptible pop and intense Achilles pain, after treatment with shock wave therapy. The assessment revealed typical rupture that was treated with surgical repair. By tracking the patient’s medical history and various treatment methods employed, the study aims to clarify the complex link between therapeutic actions and potential risks. Additionally, the case report seeks to review current literature on the adverse effects of repeated micro-trauma from percussive ultrasound therapy, suggesting that an inadequate understanding of biomechanical principles may have contributed to the unexpected complications. Ultimately, the aim of the study is to raise awareness among clinicians about appropriate treatment protocols to ensure patient safety and optimal recovery outcomes.

## Introduction

In orthopedic practice, understanding the complexities of tendon injuries is essential, particularly when evaluating modern treatment modalities such as extracorporeal shock wave therapy. Achilles tendinopathy is prevalent among Achilles disorders mainly in male athletes and physical workers with various extrinsic and intrinsic factors being the cause [1]. As for the treatment is mainly conservative with laser therapy, ultrasound, electrotherapy and shock waves combined with eccentric exercises. Patients with a prior diagnosis of Achilles tendinopathy, highlight a significant risk factor for subsequent rupture [2].

This case report highlights a case of patient suffered a notable Achilles tendon rupture following shock wave therapy, emphasizing the need for comprehensive clinical evaluation post-therapeutic interventions. This scenario raises critical questions about the cumulative effects of repeated microtrauma potentially exacerbated by rigorous percussive ultrasound treatments. The outcomes suggest a cautious approach in applying shock wave therapy, underlining the importance of well-structured rehabilitation protocols to mitigate risks associated with tendon injuries.

## Case presentation

**Figure 1:**
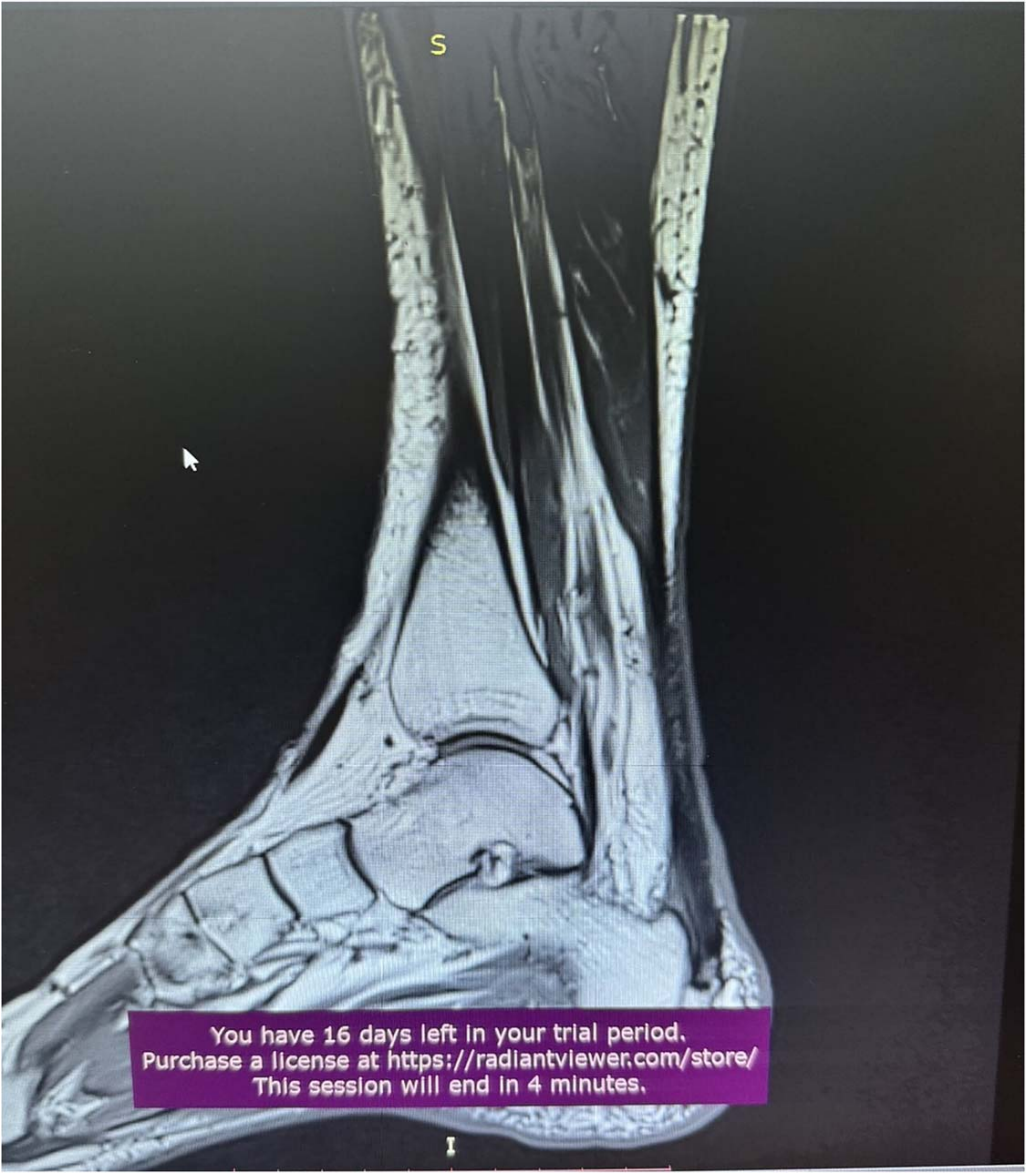
MRI of Achilles tendinopathy.

A 68-year-old female presented to the emergency department with significant discomfort localized to the Achilles tendon region. Her medical history was relatively uneventful, except for hypothyroidism and a previous diagnosis of Achilles tendinopathy (Fig. 1), that was treated conservatively with ten daily sessions of percussive ultrasound therapy. More specifically radial extracorporeal shockwave therapy (ESWT) was used, with 2.000 shockwaves at 12 Hz, which was followed by physical therapy exercises and ice therapy. Following the therapeutic sessions, the patient experienced acute symptoms, including a pronounced popping sensation and significant functional impairments.

**Figure 2:**
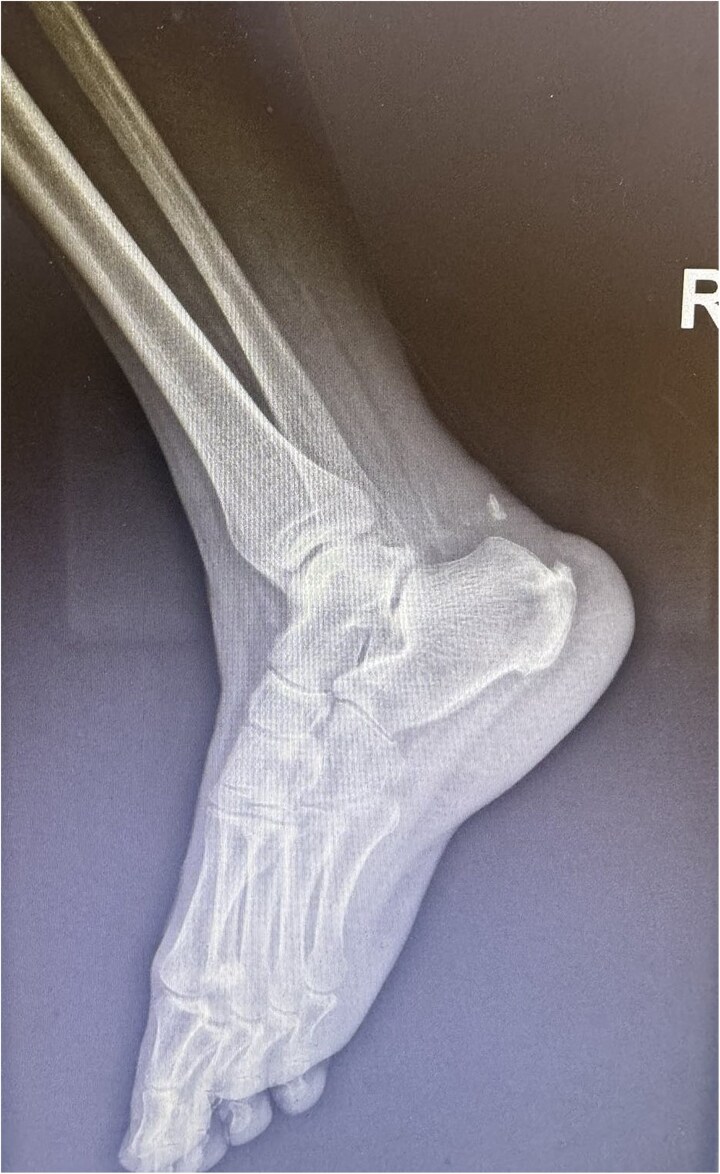
X-ray of Achilles tendon rupture with bone avulsion.

**Figure 3:**
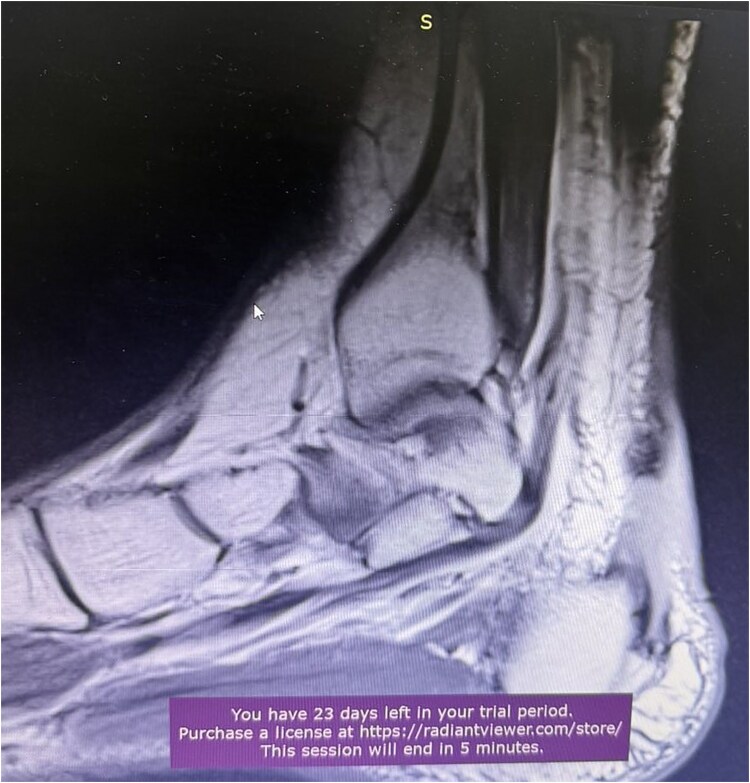
MRI of Achilles tendon rupture.

A comprehensive physical examination provided significant insights into the patient’s condition following the Achilles tendon rupture. The Thompson test was particularly noteworthy, showing an absence of plantar flexion upon calf compression, strongly indicating a complete tendon rupture. The examination further revealed a palpable gap in the Achilles tendon and increased passive dorsiflexion. Symptoms included significant weakness, difficulty walking and heel pain. Imaging studies, including X-rays (Fig. 2) and magnetic resonance imaging (MRI) (Fig. 3), confirmed these findings, showing an acute rupture with retracted tendon edges, distinguishing it from chronic condition and suggesting a possible link to the vigorous ultrasound therapy [3].

**Figure 4:**
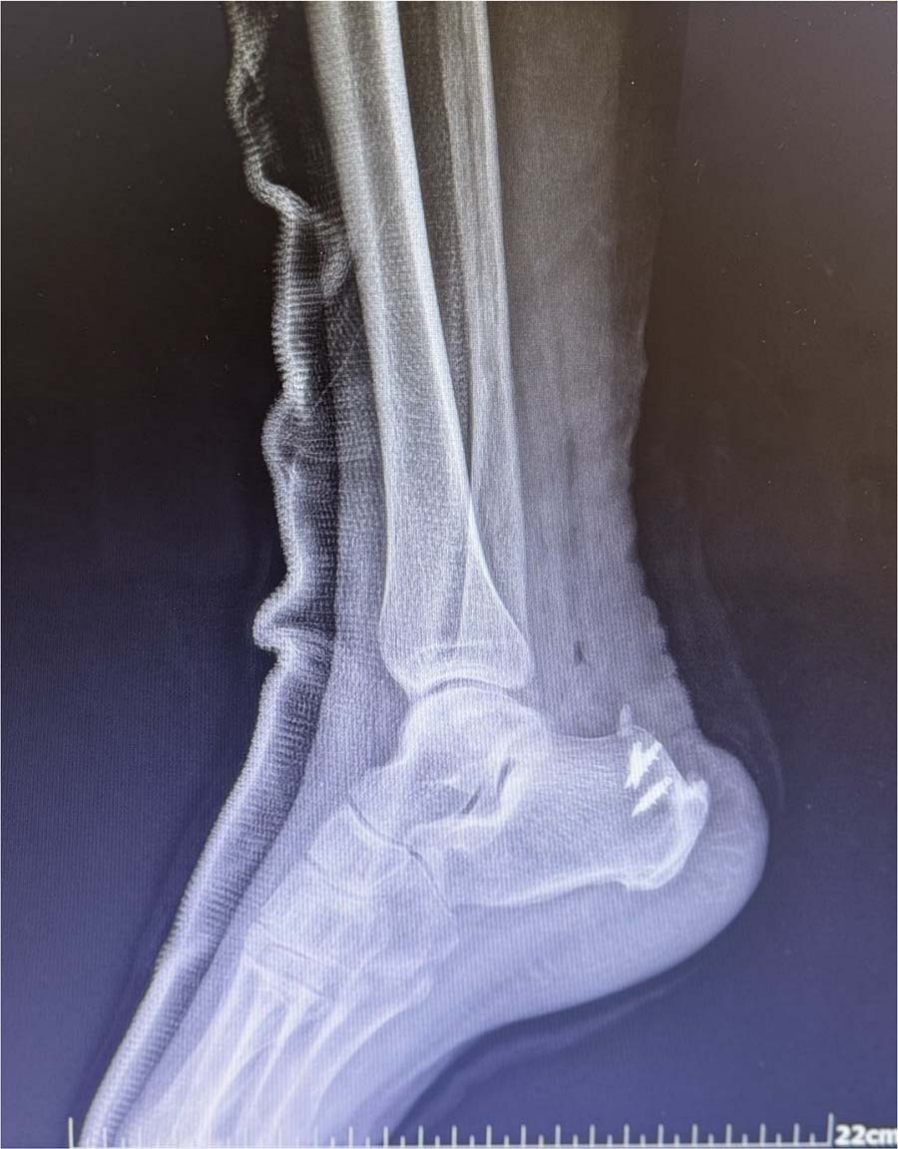
X-ray postoperatively with suture anchors.

Following the diagnosis of the Achilles tendon rupture, the initial treatment plan focused on immediate stabilization and pain management while addressing the underlying injury. After extensive clinical evaluation, surgical repair of the Achilles tendon rupture was indicated for the patient. Under general anesthesia the patient was positioned in a prone position on the operating table. The procedure included a precise incision on the posterior ankle to expose the retracted tendon segments. Using two anchors and non-absorbable sutures, the surgeon reattached the proximal tendon to the calcaneus, ensuring proper tensioning and alignment for effective healing (Fig. 4). This meticulous technique aimed to restore tendon functionality while considering the unique biomechanical stresses anticipated during rehabilitation. Postoperatively an orthosis was used, in order for the ankle to be fixed in 30° of plantarflexion and start the rehabilitation process, while also, special care was given on pain management, inflammation control, and ice therapy for the first postoperative days. Subsequently, a structured physical therapy plan was implemented to enhance functional recovery. Early range-of-motion exercises, in order to reduce stiffness and improve circulation, with a critical emphasis on avoiding excessive tendon stress during early recovery phases and, as healing progressed, rehabilitation transitioned to strengthening exercises, incorporating resistance activities tailored to the patient’s abilities and symptoms. Proprioceptive training is essential for enhancing balance and coordination that are common after Achillies tendon repair.

## Discussion

Achilles tendinopathy is an overload injury that is diagnosed clinically and it can be either at the midportion or at the insertion point of the tendon. The treatment is mostly conservative with analgetic therapy, activity modification, eccentric exercises or corticosteroid injections. Another treatment modality that is considered safe is extracorporeal shock wave therapy that is used with an exercising protocol [4], with the exception of patients with enthesophytes or Haglund’s deformity [5].

Understanding the complexities of Achilles tendon injuries requires a thorough examination of their causes, manifestations, and therapeutic interventions. Often observed in athletes and individuals engaged in repetitive high-impact activities, these injuries typically result from cumulative microtraumas [6]. Multiple factors have been proposed, including chronic overloading, medications like quinolones and corticosteroids [7], and reduced vascularity with associated heat necrosis. Other accumulative factors are age, gender, ankle and foot position, obesity and preexisting musculoskeletal disease, like Achilles tendinopathy, highlighting a significant risk factor for subsequent rupture [2]. Achilles tendon rupture accounts for 20% of all tendon ruptures and have bimodal age distribution with the second being over 60 years old [8]. Traditional symptoms, such as sudden pain, popping sensations, and functional impairments, signal potential tendon injuries, necessitating comprehensive clinical assessment and imaging to determine the extent of damage [9]. In this case, the patient underwent numerous conservative pathways, involving vigorous physical therapy, particularly percussive ultrasound. However, the intensive nature of the therapy, especially without sufficient time between each session, may have exacerbated the condition, leading to the rupture observed in clinical practice.

Shock wave therapy has emerged as a notable non-invasive method, gaining attention for its effectiveness in musculoskeletal disorders. This therapeutic approach is theorized to trigger biological reactions in affected tissues and stimulate healing by increasing neovascularization and collagen production in the affected tissue. The goal is to reduce pain and enhance functionality, ultimately restoring normal physiological activities of the tendon [10]. However, discussions about its suitability and safety persist, particularly regarding treatment guidelines and patient selection [11]. The potential for adverse outcomes arises from excessive or inappropriate use, which could inadvertently cause complications like microtrauma buildup, leading to acute injuries such as tendon ruptures, even though up to now the only adverse events observed is skin reddening and irritation without bruising and some mild discomfort depending on the density of the waves and the proximity to its treatment. Furthermore the efficacy of this treatment is based on total energy applied, the shockwave frequency, and the guidance method [12]. Thus, it is crucial for healthcare providers to conduct comprehensive assessments to optimize treatment outcomes and minimize risks associated with shock wave therapy.

Managing Achilles tendinopathy requires a well-informed approach to treatment options, including the cautious use of shock wave therapy. Continued education on biomechanical principles and proper shockwave therapy (SWT) techniques is essential to enhance patient outcomes while minimizing severe complications [13]. In this case, the patient underwent ten therapy sessions involving a specific type of ultrasound treatment to alleviate chronic pain. However, the frequency and intensity of the therapy were critical. The patient’s situation reveals gaps in current practices where aggressive methods, such as percussive ultrasound, are applied without sufficient consideration of the biomechanical effects on vulnerable tissues [14]. Additionally, the rarity of such ruptures necessitates further exploration into how shock wave therapies might incrementally cause micro-trauma, emphasizing the critical need for thorough patient histories and individualized treatment plans to prevent similar occurrences in the future.

Only one other case report of Achilles tendon rupture following extracorporeal shock wave therapy exists. The difference is that their patient suffered from chronic calcific Achilles tendinopathy of Achilles, after going through a calcaneal osteotomy for Haglund’s disease and injection of corticosteroids that can weakens even more the tendon [3]. So, our case is the first case report of Achilles tendon rupture after shock wave therapy for Achilles tendinopathy.

Future studies are needed to evaluate outcomes associated with different treatment modalities, particularly the effects of repeated treatments like percussive ultrasound, to clarify their efficacy and potential risks, including micro-injuries. Additionally, research should aim to develop more precise guidelines for rehabilitation strategies, reflecting the variability in individual tendon conditions, activity levels, and healing capacities [15].

## Conclusion

This case report highlights significant findings where the patient progressed from Achilles tendinopathy to a complete rupture due to ongoing microtrauma associated with percussive ultrasound therapy due to the high diversity of application methodologies. Emphasizes the need for a comprehensive approach to physical therapy, integrating appropriate treatment planning that respects tissue integrity, rather than relying solely on high-impact techniques. These findings reinforce the importance of meticulous monitoring and individualized intervention approaches in managing tendon injuries to prevent severe outcomes.

## Data Availability

Data generated during the study are subject to a data sharing mandate and available in a public repository that does not issue datasets with DOIs.
